# A case of recurrent depressive disorder presenting with Alice in Wonderland syndrome: psychopathology and pre- and post-treatment FDG-PET findings

**DOI:** 10.1186/s12888-017-1314-2

**Published:** 2017-04-27

**Authors:** Tatsushi Yokoyama, Tsuyoshi Okamura, Miwako Takahashi, Toshimitsu Momose, Shinsuke Kondo

**Affiliations:** 1 Department of Neuropsychiatry, Graduate School of Medicine, University of Tokyo, 7-3-1 Hongo, Bunkyo-ku, Tokyo 113-8655 Japan; 2 Department of Radiology, Graduate School of Medicine, University of Tokyo, 7-3-1Hongo, Bunkyo-ku, Tokyo 113-8655 Japan

**Keywords:** Alice in Wonderland syndrome, Recurrent depressive disorder, Psychotic depression, Fluorodeoxyglucose positron emission tomography, High-order brain function, Case report

## Abstract

**Background:**

Alice in Wonderland syndrome (AIWS) is a rare neuropsychiatric syndrome that typically manifests in distortion of extrapersonal visual image, altered perception of one’s body image, and a disturbed sense of the passage of distance and time. Several conditions have been reported to contribute to AIWS, although its biological basis is still unknown. Here, we present the first case demonstrating a clear concurrence of recurrent depressive disorder and AIWS. The clinical manifestations and pre- and post-treatment fluorodeoxyglucose positron-emission tomographic (FDG-PET) images provide insights into the psychopathological and biological basis of AIWS.

**Case presentation:**

We describe a 63-year-old Japanese male who developed two distinct episodes of major depression concurrent with AIWS. In addition to typical AIWS perceptual symptoms, he complained of losing the ability to intuitively grasp the seriousness of news and the value of money, which implies disturbance of high-order cognition related to estimating magnitude and worth. Both depression and AIWS remitted after treatment in each episode. Pre-treatment FDG-PET images showed significant hypometabolism in the frontal cortex and hypermetabolism in the occipital and parietal cortex. Post-treatment images showed improvement of these abnormalities.

**Conclusions:**

The clinical co-occurrence of depressive episodes and presentation of AIWS can be interpreted to mean that they have certain functional disturbances in common. In view of incapacity, indifference, devitalization, altered perception of one’s body image, and disturbed sense of time and space, the features of AIWS analogous to those of psychotic depression imply a common psychopathological basis. These high-order brain dysfunctions are possibly associated with the metabolic abnormalities in visual and parietotemporal association cortices that we observed on the pre- and post-treatment FDG-PET images in this case, while the hypometabolism in the frontal cortex is probably associated with depressive symptoms.

## Background

Alice in Wonderland syndrome (AIWS) is a rare neuropsychiatric syndrome, which Todd [1] named after the character created by Lewis Carroll. Collective manifestations of AIWS include distortion of extrapersonal visual image (micropsia, macropsia, metamorphopsia, teleopsia, and pelopsia), altered perception of one’s body image, and a disturbed sense of the passage of distance and time [2].

Many conditions have been reported to contribute to AIWS, and Lanska et al. [2] indicates that viral infection and migraine are the two most commonly identified causes, occurring preferentially in young individuals. To the best of our knowledge, only two reports to date have associated AIWS with a depressive disorder [3, 4], neither of which clearly showed the concurrent nature of major depressive episodes and AIWS.

Evidence from a number of studies using fluorodeoxyglucose positron emission tomography (FDG-PET) indicates that specific cortical regions, primarily in the frontal cortex, are related to mood disorders. However, the results from imaging studies have not yielded a clear story, and many conflict with each other [5–7]. One of the more consistent findings with regard to depression, is that hypometabolism in the frontal cortex is characteristic in patients with depressive episodes, and can be partially reversed by successful treatment. In contrast, the biological basis of AIWS is even less clear, although a few studies have made attempts studying it using functional brain imaging [8–11].

Here, we present a case of an older patient whose recurrent depressive episodes and AIWS emerged and remitted simultaneously with successful treatment with antidepressants, antipsychotics, and electroconvulsive therapy (ECT). This is the first report showing the concurrent and recurrent nature of, depressive episodes and AIWS, and the strong association between the two. The clinical manifestations and pre- and post-treatment FDG-PET images provide insights into the psychopathological and biological basis of AIWS.

## Case presentation

Here, we report the case of a 63-year-old Japanese man with no medical or psychiatric history, except for type-2 diabetes mellitus and essential hypertension. He had no previous history of psychotropic drug use, including antidepressants and antipsychotics. Additionally, he had no developmental abnormalities or neurodevelopmental disorders. He held a steady job from college graduation until retirement age, and his wife described his premorbid personality as dependable, sociable, and patient. He had no family history of psychiatric disorders, migraine, or epilepsy.

One year before his first admission to an inpatient psychiatric unit, he started experiencing mild depressive moods and fatigue that did not disrupt his day-to-day functioning. Two months before the first hospital admission, he began complaining about typical AIWS symptoms, including micropsia, altered perception of his body image, and a disturbed sense of the passage of distance and time. All sorts of objects in his environment, such as buildings and cars, looked extremely small to him. He gave up driving because cars looked so small that he lost his sense of speed and distance in relation to the cars around him. Nearby objects also looked very small, with the single exception of pill strips that he had difficulty opening. Moreover, even though he knew it was not possible, he felt as if he could ‘step over’ long distances in a flash, such as the 50 km from his suburban town to the center of Tokyo. Additionally, he felt that days passed extremely quickly, as if in a single moment. He also sometimes felt his body was slightly enlarged or shrunken compared with normal. These AIWS symptoms persisted all day long during the depressive episodes.

In addition to the typical AIWS symptoms described above, he also complained of disturbances in high-order cognition. For instance, he said, “I cannot sense how important the news is. For example, when I see news about a serial murder on television, I can understand intellectually how sad it is, but I cannot realize it emotionally”. Similarly, he said, “I cannot appreciate the value of money. Even if there were a ¥10,000 bill in front of me, I wouldn’t care about it because I can’t realize how much value it would have”. Although his bowel movements and urination were normal, he complained of a decreased urge to defecate and urinate.

The depressed mood, loss of interest and pleasure, psychomotor retardation, fatigue, and reduced concentration gradually worsened. He was referred to a neurologist. Organic causes were ruled out as follows: his blood-sugar level and blood pressure were well controlled with insulin injections and oral medications; he was a non-drinker, had no history of head trauma, and took no medications associated with adverse reactions that could mimic depression, such as beta-blockers and cimetidine. Neurological examinations and laboratory tests including endocrine evaluations and an HIV test, electroencephalography, and brain magnetic resonance imaging (MRI) detected no abnormalities. He was then referred to a psychiatrist. After confirming that he was not experiencing a manic episode, was not using illicit drugs, and had not experienced any recent stressful life events, he was diagnosed as having a severe depressive episode with AIWS. His condition worsened to the degree that he could not continue working despite taking paroxetine, and he was hospitalized for the first time.

At this first admission, he was bed-ridden all day because of severe depressive symptoms. Administration of amitriptyline (75 mg/day) and perphenazine (6 mg/day) induced gradual improvement of depressive and AIWS symptoms. He was discharged on day 47 after he had remitted almost completely from the depressive episode, with the exception of easily becoming fatigued and waking at night. At that time, he was also completely remitted from AIWS. His day-to-day functioning returned to normal, and his work and life continued as they had before the episode began.

Three years after discharge, he relapsed into another major depressive episode, again simultaneously presenting with AIWS. The symptoms worsened despite the use of amitriptyline (50 mg/day) and aripiprazole (6 mg/day) in the outpatient clinic. The Visual Perception Test for Agnosia detected nothing abnormal. His thoughts became stunted and he became very inactive, lying in bed all day. He continuously refused inpatient treatment because he delusionally believed he was too poor. Upon the strong recommendation from his family, 8 months after this recurrence, he was admitted to the hospital with recurrent severe depressive symptoms and AIWS at the age of 67 years.

At this second admission, he was alert and oriented, but had prolonged speech latency and spoke in a slow and quiet manner without making eye contact. His face was unshaven and he did not smile. Dementia was ruled out as a plausible cause of his symptoms for the following reasons: 1) his Mini-Mental State Examination (MMSE) score was 28/30 during this depressive episode, 2) he made a complete recovery from the observed reduction in concentration and processing speed after treatment of the first episode, 3) he exhibited no other signs of recognizable cognitive decline such as impaired executive function, learning, memory, language, or social recognition, and 4) he did not exhibit any typical symptoms of common dementia subtypes, such as amnesia, fluctuating cognition, visual hallucinations, extrapyramidal symptoms, or behavioral symptoms. Evidence of depressive symptoms and AIWS was comparable between the first and second episodes. He scored 30/63 on the Beck Depression Inventory-II (BDI-II), indicating severe depression. An ophthalmologist confirmed no eye/visual abnormalities with the exception of bilateral cataracts. Pre-therapy FDG-PET was performed as described below. After 2 weeks of maprotiline (75 mg/day) had no effect, twice-weekly ECT, duloxetine (60 mg/day) and mirtazapine (45 mg/day) were administered. He remitted completely from AIWS and almost completely from the depressive episode after 12 ECT sessions, except for a mild reduction in concentration. He scored 12/63 on the BDI-II, which also indicated significant recovery from depression. He was discharged after 75 days, just after post-therapeutic FDG-PET was performed.

### FDG-PET acquisition, visual inspection, and statistical analysis

We obtained the pre- and post-treatment FDG-PET images of the brain during the second admission. The patient was kept at rest in supine posture with a blinder in a quiet and dim room from 10 min before each PET examination until the end of the scan. Scans were recorded with a PET scanner (Advance NXi; GE Medical Systems, Milwaukee, WI, USA) 45 min after the injection of 296 MBq FDG.

**Fig. 1:**
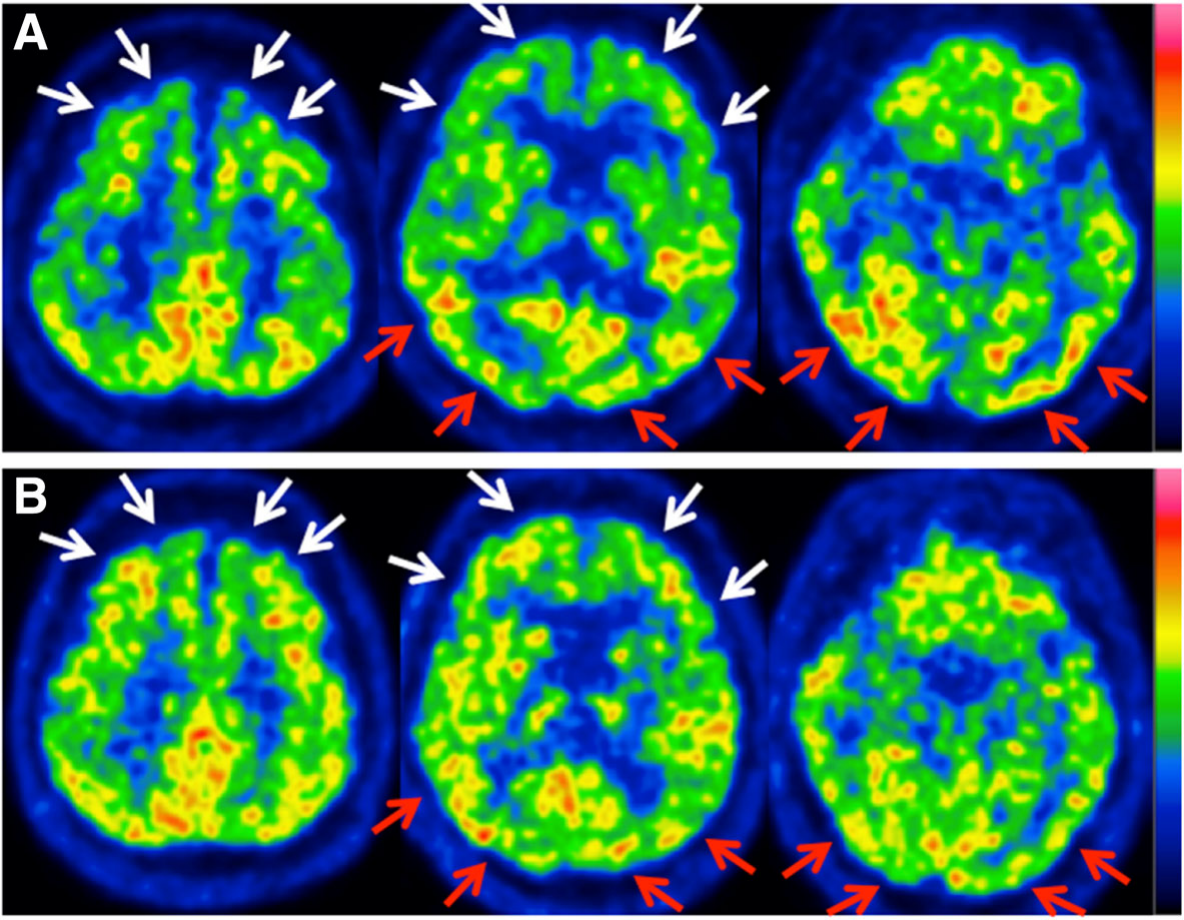
Axial pre- and post-treatment brain FDG-PET images. The color scale ranges from zero to the maximum value within the brain. **a** Pre-treatment images show hypometabolism in the bilateral frontal cortex (*white arrows*) and relative hypermetabolism in the bilateral occipital and parietal cortex (*red arrows*). **b** Post-treatment images show slight normalization of these abnormalities

Upon visual inspection, the pre-treatment FDG-PET images depicted moderate hypometabolism in the frontal cortex and relative hypermetabolism in the occipital and parietal cortices (Fig. 1a). These abnormalities improved slightly after treatment (Fig. 1b).

**Fig. 2:**
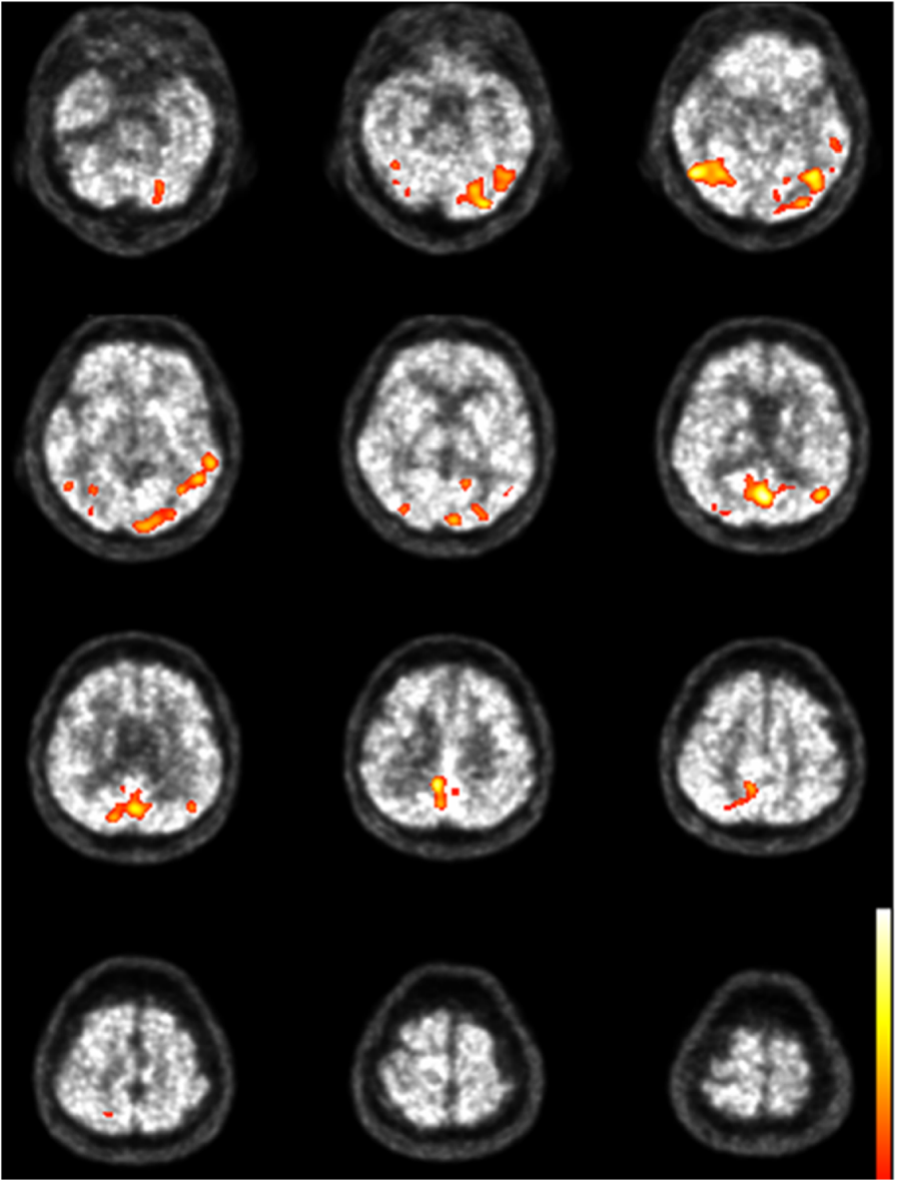
Within-subject comparison between pre- and post-treatment in our patient. The colored areas indicate significant decreases in metabolism from pre- to post-treatment, with the color scale (z score) ranging from 2 to 5

Statistical analysis was performed in the following steps: (1) morphological co-registration between pre-and post-treatment FDG-PET; (2) normalization of voxel values to the global mean voxel counts using proportional scaling; (3) subtraction of pre-treatment from post-treatment images to obtain pre-post difference images; (4) mean and standard deviations of voxel values were calculated for the difference images; and (5) identification of area with statistically significant difference, using a cutoff value of z > 2 and extent threshold k > 200. These methods are part of the standard process for subtracting ictal single photon emission computed tomography (SPECT) coregistered to MRI (SISCOM), which is generally used for comparing ictal and interictal SPECT images in epileptic patients [12]. The statistical analysis showed that metabolism decreased after treatment in the posterior half of the cerebral cortex, including the posterior part of the bilateral temporal cortex, the occipital cortex, the inferior part of parietal cortex, precuneus, and posterior cingulate cortex (Fig. 2). No area showed statistically significant increases in metabolism after treatment.

## Discussion

### Synchronicity of depressive episodes and AIWS

In this case study, we described an older patient presenting with two distinct major depressive episodes, both of which occurred simultaneously with episodes of AIWS. Both sets of symptoms remitted completely after standard treatment for psychotic depression (i.e., an antidepressant plus an antipsychotic, or ECT), and we excluded the potential influence of dementia and other organic causes. This is the first report that clearly shows a link between recurrent depressive disorder and AIWS. The clinical co-occurrence of depressive episodes and AIWS can be interpreted as arising from common functional disturbances. Below we elaborate on the relationship between AIWS and severe depression from the viewpoints of psychopathology and of neuroimaging.

### AIWS presenting in the course of psychotic depression

Lanska et al. reported that a variety of conditions contribute to AIWS, including infection, migraine, toxic encephalopathy, major depression, epileptic seizures, medications, and stroke [2]. However, to date, only two case reports have shown an association between depressive episodes and AIWS [3, 4]. Mizuno et al. reported a 54-year-old man who exhibited depressive symptoms with AIWS (metamorphopsia, disturbed sense of passage of time, and distortion of body image). In that case, AIWS disappeared 2 days after admission despite no improvement in depressive symptoms [3]. Bui et al. reported a 74-year-old man who showed severe depressive symptoms with persecutory and somatic delusions (his stools being contaminated) and AIWS (he thought his hands and feet were shorter than usual), all of which remitted completely with ECT [4]. Importantly, both cases presented with psychotic depression during the clinical course. In Mizuno et al., the patient developed a delusion of culpability and in Bui et al., the patient developed persecutory and somatic delusions. Our patient also transiently developed delusions (he believed he was poor) during the depressive episode. The clinical presentation of these three cases indicates an association between AIWS and psychotic depression in particular.

### Psychopathological similarities between AIWS and psychotic depression

Patients with psychotic depression tend to regard themselves as being useless, or consider everything meaningless. They also claim to lose a grasp on proper emotions and visceral sensations. Stanghellini et al. extensively reviewed the psychopathology of depression and described the qualitative features of experience in patients with psychotic depression to include incapacity, indifference, timelessness, and bodily devitalization [13]. Failure to sense the significance or magnitude of external facts or objects, such as the difficulty with news and money seen in our patient, can be viewed as being part of the incapacity or indifference categories. Being unable to estimate or sense one’s own urge to urinate or make bowel movements can also be explained by indifference or devitalization. According to Stanghellini et al. constriction of time and body experiences, as well as shrinking and extension of space, occur in major depression. Perceptual alterations in space, time, and body seem to be notably analogous to that of AIWS. Thus, we argue that comorbidity of AIWS and psychotic depression in our case was not by chance, but occurred because of a common underlying brain dysfunction.

### Interpretation of FDG-PET results

The frontal cortex hypometabolism in our case was probably related to the depressive symptoms (Fig. 1). Hosokawa et al. reported that euthymic patients exhibited fewer areas with significantly low metabolism than did depressed patients in the cross-sectional study [5]. In our case, the decreased metabolism of bilateral frontal cortex and anterior cingulate cortex during the second depressive episode improved after treatment, although these alterations were not statistically significant. These results are consistent with many studies demonstrating that metabolism in the frontal cortex decreases in patients with depressive episodes and can be partially reversed by treatment [5, 6].

The significant metabolic abnormalities and alterations in the posterior half of the cerebral cortex are the main characteristics of our case (Figs. 1, 2). These regions include the primary visual cortex, precuneus, posterior cingulate, and temporal, parietal, and extrastriate cortices. The abnormalities in these association cortices possibly correspond to positive symptoms in our case, such as delusions of poverty or experiencing alterations in space, time, and body, which are not simply perceptual, but also include high-order cognitive disturbances. The parieto-occipital hypermetabolism in our case might also be associated with a common underlying abnormality that occurs in both psychotic depression and AIWS, although this should be explored in future studies.

## Conclusion

We present the first case demonstrating a clear relationship between recurrent depressive disorder and AIWS. The co-occurrence and similarities in clinical manifestations between AIWS and psychotic depression imply a common psychopathological basis. The metabolic abnormalities seen in high-order brain regions on FDG-PET images suggest a biological basis of AIWS and psychotic symptoms of depression. Careful analysis of psychiatric symptoms comparing with metabolic changes using FDG-PET provides new insights into higher-order involvements, not only primary visual perception, in AIWS and psychotic depression.

## Abbreviations

AIWS: Alice in Wonderland syndrome
BDI-II: Beck Depression Inventory-II
ECT: Electroconvulsive therapy
FDG-PET: Fluorodeoxyglucose positron emission tomography
MMSE: Mini-Mental State Examination
MRI: Magnetic resonance imaging
SISCOM: Subtraction ictal SPECT coregistered to MRI
SPECT: Single photon emission computed tomography
